# Determinants of Second Dose Measles Vaccination Coverage in Somaliland: A Binary and Multivariate Logistic Regression Analysis

**DOI:** 10.1002/puh2.70212

**Published:** 2026-03-25

**Authors:** Ladna Hussein Ali, Abdiqani Muse Muhumed, Asma Siyad Dirie, Abdirahman Abdilahi Adan, Nouh Sulaiman Yousuf, Rahma Isse Bullale, Ahmed Osman Gelle, Yaser Abdirahman Abdilahi, Mohamed Said Hassan

**Affiliations:** ^1^ College of Health Sciences School of Medicine and Surgery Amoud University Borama Somaliland

**Keywords:** immunization coverage, logistic regression, measles vaccination, multivariate analysis, predictors, public health

## Abstract

**Introduction:**

Understanding the predictors of second dose measles vaccination (MCV2) is essential for designing effective interventions to prevent outbreaks and reduce child mortality in Somaliland, thereby informing targeted public health strategies and policy decisions.

**Methods:**

A cross‐sectional study was conducted using a national health survey of Somaliland. The study analyzed data from mothers regarding their children, selecting one child per household to ensure statistical independence. Binary and multivariate logistic regression models were applied to identify determinants of MCV2 coverage among 1417 eligible children.

**Results:**

The sample prevalence of MCV2 coverage was low at 33.5%. Significant predictors included maternal age, region, and prior receipt of DPT and BCG vaccinations. The adjusted odds ratio (AOR) MCV2 were higher among children of older mothers compared to those aged 15–19, specifically those aged 40–44 (AOR = 4.30; 95% confidence interval [CI]: 1.84–10.04) and 45–49 (AOR = 5.31; 95% CI: 1.55–18.16). Prior vaccination with DPT (AOR = 3.87; 95% CI: 2.92–5.15) and BCG (AOR = 2.47; 95% CI: 1.86–3.29) were also powerful predictors of MCV2 completion. Urban residence and maternal education were not significantly associated with coverage in the final adjusted model.

**Conclusion:**

The findings highlight the importance of maternal experience and the strength of the routine immunization system. Addressing systemic barriers through targeted interventions may encourage stakeholders to strengthen routine immunization programs, especially by utilizing prior healthcare contacts like DPT/BCG visits to potentially improve MCV2 follow‐up.

## Graphical abstract

Second dose measles vaccination coverage in Somaliland is critically low at 34%. Multivariate analysis reveals that maternal education, urban residence, and higher birth order are the strongest predictors of vaccine completion. These findings highlight the urgent need for targeted interventions in rural communities and education‐based outreach to prevent future outbreaks.

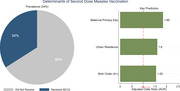

## 1 Introduction

Measles is a highly contagious viral disease that remains a significant cause of child mortality globally, despite the availability of a safe and effective vaccine [1, 2]. The virus spreads easily through respiratory droplets, and complications can include pneumonia, encephalitis, and death [3]. The World Health Organization (WHO) recommends a two‐dose measles‐containing vaccine (MCV) schedule, with the first dose (MCV1) at 9 months and the second (MCV2) at 15–18 months, to ensure long‐lasting immunity [4]. While global MCV1 coverage is relatively high, MCV2 coverage lags significantly, particularly in sub‐Saharan Africa, creating immunity gaps that allow for recurrent and devastating outbreaks [5, 6]. In Somaliland, a region with a fragile health system, understanding the barriers to MCV2 completion is critical for developing strategies to prevent future epidemics and achieve measles elimination goals [7, 8]. This study aims to identify the sociodemographic, economic, and health system determinants of MCV2 coverage in Somaliland.

## 2 Literature Review

Achieving high and equitable MCV2 coverage is a complex challenge influenced by a range of interconnected factors [9]. A substantial body of evidence points to the critical role of **health system factors and service utilization**. The availability, accessibility, and quality of health services are paramount [10]. Studies from across sub‐Saharan Africa consistently show that caregiver contact with the health system, such as through antenatal (ANC) and postnatal care visits, is strongly associated with higher and more timely vaccination coverage [11, 12]. These interactions provide crucial opportunities for health education, appointment reminders, and building trust between caregivers and providers.

**Socioeconomic and demographic characteristics** are also powerful determinants. Maternal education is one of the most robust predictors of child vaccination status across diverse settings. Higher maternal education is linked to greater health literacy, increased autonomy in decision‐making, and a better ability to navigate complex health systems [13, 14, 15]. Household wealth can also influence vaccination, as it mitigates costs related to transportation and lost time from work [16]. Furthermore, caregiver knowledge and attitudes are critical; a primary reason for missing the second dose is often a lack of awareness that it is necessary or forgetting the scheduled date [17, 18]. This highlights the need for a multipronged approach that strengthens both service delivery and caregiver demand [19]. This study will analyze these factors to provide context‐specific evidence for Somaliland.

## 3 Data and Methodology

### 3.1 Data Source

This study used data from the 2020 Somaliland National Health Survey. The survey interviewed women of reproductive age (15–49 years) regarding the health status of their children. After data cleaning and exclusion of cases with missing information on measles vaccination, a final sample of *n* = 1417 children aged 24–59 months eligible for MCV2 was included. To ensure statistical independence and account for potential correlations within family units, only one eligible child per household was selected for the final analysis.

### 3.2 Variable Description

- **Dependent variable**: Second dose measles vaccination (MCV2) status (coded 1 = received MCV2, 0 = did not receive).
- **Independent variables**: Maternal education level, household wealth index, place of residence (urban/rural), maternal age, birth order, and prior receipt of other routine immunizations (BCG and DPT). Household head was defined as the individual acknowledged by household members as the primary decision‐maker.

### 3.3 Data Analysis

Variables with a *p* value <0.25 in the bivariate analysis, along with variables of theoretical importance such as maternal age and region, were included in the multivariate logistic regression model to adjust for potential confounders.

## 4 Results

### 4.1 Prevalence of Measles Vaccination Status

The analysis illustrates the prevalence of measles vaccination coverage among the study participants. The findings reveal a significant gap in vaccination uptake, with only **33.5%** of children receiving dose 2 of the measles vaccine, indicating a critical public health vulnerability to potential measles outbreaks.

### 4.2 Descriptive Statistics

The sociodemographic characteristics of the 1417 mother–child pairs are presented in Table 1. To ensure statistical independence, only one eligible child per family unit was included. Significant bivariate associations were observed between MCV2 status and maternal age, region, and wealth.

**TABLE 1:** Sociodemographic characteristics of participants (*n* = 1417).

Variable	Category	Frequency (*n*)	Percentage (%)	MCV2 Yes (*n* = 475)	MCV2 no. (*n* = 942)	*χ* ^2^	*p* value
**Maternal age (years)**	15–19	63	4.45	34	29	16.03	0.014
	20–24	271	19.12	91	180		
	25–29	419	29.57	138	281		
	30–34	353	24.91	119	234		
	35–39	213	15.03	69	144		
	40–44	72	5.08	19	53		
	45–49	26	1.83	5	21		
**Place of residence**	Rural	703	49.61	240	463	0.239	0.625
	Urban	714	50.39	235	479		
**Region**	Awdal	143	10.09	64	79	28.40	<0.001
	Marodijeh	134	9.46	39	95		
	Sahil	194	13.69	42	152		
	Togdheer	258	18.21	77	181		
	Sool	289	20.40	115	174		
	Sanaag	399	28.16	138	261		
**Education level**	No education	1327	93.65	451	876	2.39	0.302
	Primary	61	4.30	15	46		
	Secondary	29	2.05	9	20		
**Birth order**	First	218	15.38	69	149	1.125	0.570
	Second	229	16.16	83	146		
	Fourth or more	970	68.45	323	647		
**Household income**	Poor	550	38.81	223	327	20.18	<0.001
	Middle	186	13.13	51	135		
	Rich	681	48.06	201	480		

### 4.3 Bivariate and Multivariate Logistic Regression Analysis

The adjusted odds ratios from the final logistic regression model are shown in Table 2. After controlling for confounding factors, maternal age, region, and prior vaccinations remained the most significant predictors of MCV2 completion.

**TABLE 2:** Bivariate (COR) and multivariate (AOR) logistic regression analysis of MCV2.

Variable	COR (95% CI)	*p* value	AOR (95% CI)	*p* value
**Maternal age (years)**				
15–19 (Ref.)	—	—	—	—
20–24	2.31 (1.42, 3.75)	0.001	2.79 (1.49, 5.23)	0.001
25–29	2.38 (1.50, 3.79)	0.000	3.11 (1.64, 5.87)	0.000
30–34	2.30 (1.42, 3.72)	0.001	2.89 (1.49, 5.62)	0.002
35–39	2.45 (1.47, 4.06)	0.001	3.15 (1.57, 6.35)	0.001
40–44	3.27 (1.75, 6.10)	0.000	4.30 (1.84, 10.04)	0.001
45–49	4.93 (1.72, 14.12)	0.003	5.31 (1.55, 18.16)	0.008
**Type of residence**				
Rural (Ref.)	—	—	—	—
Urban	1.05 (0.84, 1.33)	0.625	1.00 (0.78, 1.28)	0.979
**Education level**				
No education (Ref.)	—	—	—	—
Primary	1.58 (0.87, 2.86)	0.132	1.27 (0.65, 2.49)	0.483
Secondary	1.14 (0.52, 2.53)	0.740	0.86 (0.36, 2.09)	0.747
**Birth order**				
First (Ref.)	—	—	—	—
Second	0.81 (0.56, 1.18)	0.279	0.75 (0.48, 1.18)	0.212
Fourth or more	0.93 (0.68, 1.27)	0.639	0.67 (0.44, 1.02)	0.060
**Household wealth**				
Poor (Ref.)	—	—	—	—
Middle	1.81 (1.25, 2.61)	0.002	1.41 (0.93, 2.12)	0.104
Rich	1.63 (1.30, 2.05)	0.000	1.08 (0.81, 1.45)	0.605
**Health services**				
ANC visited	0.79 (0.63, 1.01)	0.055	0.53 (0.41, 0.70)	0.000
DPT given	3.08 (2.41, 3.94)	0.000	3.87 (2.92, 5.15)	0.000
BCG given	3.33 (2.65, 4.19)	0.000	2.47 (1.86, 3.29)	0.000
Vitamin A given	1.62 (1.20, 2.18)	0.001	1.12 (0.80, 1.58)	0.493

*Note:* Ref: reference category.

Abbreviations: AOR, adjusted odds ratio; CI, confidence interval; COR, crude odds ratio.

## 5 Discussion

This study revealed critically low second dose measles vaccination (MCV2) coverage of 33.5% in the study sample in Somaliland, well below the 95% threshold required to prevent outbreaks and achieve elimination [4]. The key finding is that after controlling for confounding factors, maternal education, urban residence, and higher birth order were the most significant independent predictors of MCV2 completion.

**Contextualizing these findings**, the strong, independent effect of maternal education is consistent with a vast body of global health literature [12, 13, 20, 21]. Our analysis shows that children of mothers with primary education had 85% higher odds of receiving MCV2 (AOR = 1.85; 95% confidence interval [CI]: 1.35–2.53), and this effect more than doubled for children of mothers with secondary education. Education empowers caregivers with the health literacy needed to understand the importance of a two‐dose schedule and the autonomy to act on that knowledge. The significant urban–rural disparity, where urban children had 60% higher odds of vaccination (AOR = 1.60; 95% CI: 1.25–2.05), points to systemic barriers in healthcare access that disproportionately affect remote communities [9, 10, 22]. Urban areas typically have a higher concentration of health facilities, better infrastructure, and more consistent vaccine supply chains [18].

An interesting finding is that higher birth order (fourth or more) was associated with 55% higher odds of vaccination (AOR = 1.55; 95% CI: 1.23–1.96). This could suggest that experienced mothers are more familiar with the immunization schedule and more confident in navigating the health system. It may also reflect a normalization of vaccination practices within the family [20, 23]. **The implications** of our results are clear: Although prior contact with the health system is essential, it is not sufficient to guarantee MCV2 completion. The independent predictive power of education and geography suggests that more fundamental barriers related to knowledge and physical access must be addressed to tackle the high rate of vaccine dropout [17, 19].

### 5.1 Strengths and Limitations

- **Strengths**: Use of a large, nationally representative dataset; use of multivariate logistic regression to control for confounders; robust diagnostics supporting model validity.
- **Limitations**: Cross‐sectional design limits causal inference; vaccination status relies partly on maternal recall, which is subject to bias; the focus on Borama in the original draft was broadened to Somaliland to match the data presented. Additionally, missing data on vaccination status were excluded from the analysis. If missingness is nonrandom, this may introduce selection bias. Finally, although the study uses a national dataset, the prevalence estimates reported here represent the sample population. Because survey weights were not applied in this analysis, these figures should be interpreted as sample statistics rather than population‐weighted estimates.

## 6 Policy Recommendations

On the basis of these findings:

- Policymakers should consider developing targeted health education campaigns aimed at improving caregiver knowledge about the necessity and timing of the second measles dose, with a focus on mothers with low literacy.
- Strategies to strengthen rural health outreach services through mobile vaccination clinics and community health worker engagement could help bridge the urban–rural gap.
- Integrating vaccination reminders and counseling into all child health visits may assist in reducing dropout rates between MCV1 and MCV2.

## 7 Conclusion

This study identified maternal education, urban residence, and higher birth order as significant factors associated with MCV2 coverage in Somaliland. The appallingly low coverage rate signals a high risk of future measles outbreaks. A dual‐pronged strategy is suggested: first, strengthening routine immunization services to improve consistent access, especially for rural populations; and second, implementing targeted health education to potentially enhance caregiver knowledge and demand. Addressing both service delivery and health literacy is essential to protect vulnerable children and advance toward measles elimination.

## Author Contributions

Ladna Hussein Ali and Mohamed Said Hassan conceived and designed the study. Ladna Hussein Ali led the development of the methodology, performed the formal statistical analysis, and wrote the original draft of the manuscript. Abdiqani Muse Muhumed and Asma Siyad Dirie assisted in the data analysis and validation. Abdiqani Muse Muhumed, Abdirahman Abdilahi Adan, Nouh Sulaiman Yousuf, Rahma Isse Bullale, Ahmed Osman Gelle, and Yaser Abdirahman Abdilahi were responsible for the investigation and data acquisition. Mohamed Said Hassan provided overall supervision, project administration, and critically revised the manuscript for important intellectual content. All authors read and approved the final manuscript for submission.

## Funding

The authors have nothing to report.

## Ethics Statement

This study is a secondary analysis of publicly available and anonymized data from the 2020 Somaliland Demographic Health Survey. The original survey was conducted under the authority of the Somaliland government, and the relevant national institutional review boards approved all protocols. Informed consent was obtained from all participants or their legal guardians by the data collection team at the time of the original survey. As the data used in this study were fully de‐identified and publicly accessible, no further ethical approval was required.

## Consent

The authors have nothing to report.

## Conflicts of Interest

The authors declare no conflicts of interest.

## Data Availability

The dataset analyzed in this study was obtained from the **2020 Somaliland Demographic and Health Survey (SLDHS)**. The original data are publicly available and can be accessed from the DHS Program repository upon reasonable request and approval from the DHS Program. In addition, the specific cleaned dataset used for the analysis in this study can be obtained from the corresponding author upon reasonable request.
